# Supplementary material for: Virtual Screening and ADMET Prediction to Uncover the Potency of Flavonoids from Genus Erythrina as Antibacterial Agent through Inhibition of Bacterial ATPase DNA Gyrase B
Source: Molecules. 2023 Dec 8;28(24):8010. doi: 10.3390/molecules28248010 (PMC10745610; doi:10.3390/molecules28248010)
Supplement: Supplementary file 1 [file molecules-28-08010-s001.zip › molecules-2595116-supplementary.pdf]

Supplementary Material

Table S1. Binding affinity.

Compound Code	Compounds	Binding Affinity
	Co-crystal ligand	-8.853
	ATP	-7.498
14	Apigenin-7-O-rhamnosyl-6-C-glucoside	-9.615
20	Kaempferol-3-O-(2′′-O-β-ᴅ-glucopyranosyl-6′′-O-α-L-rhamnopyranosyl-β-ᴅ-glucopyranoside)	-9.599
74	Lonchocarpol C	-9.489
131	Eryzerin C	-9.411
70	2(S)-5,7-Dihydroxy-[(5′′,6′′:3′,4′)- (2′′,2′′-dimethylpyrano)-(5′′′,6′′′:5′,6′)]-(2′′′,2′′′- dimethylpyrano)flavanone	-9.373
64	Sigmoidin E	-9.352
94	(2S)-5,7-Dihydroxy-5′-prenyl-2′′-(4′′-hydroxyisopropyl)-dihydrofurano[1′′,3′′:3′,4′] flavanone	-9.287
210	Isolupabigenin	-9.244
88	2(S)-5,7-Dihydroxy-5′-prenyl- [2′′,2′′-(3′′-hydroxy)- dimethylpyrano]-(5′′,6′′:3′,4′) flavanone	-9.228
7	Neocyclomorusin	-9.208
79	Fuscaflavanones B	-9.184
96	(2S)-5,7,5′-Trihydroxy-2′′-(4′′- hydroxyisopropyl)-dihydrofurano [1′′,3′′:3′,4′]flavanone	-9.149
52	Iespedezaflavanone B (Euchrestaflavanone A)	-9.124
130	Eryzerin D	-9.124
16	Vicenin-2	-9.097
178	5,3′-Dihydroxy-4′-methoxy-5′-γ,γ-dimethylallyl-2′′,2′′-dimethylpyrano[5,6:6,7] isoflavanone	-9.096
98	(2S)-5,7,5′-Trihydroxy-2′′-(4′′- hydroxyisopropyl)-3′′-hydroxy-dihydrofurano[1′′,3′′:3′,4′] flavanone	-9.094
75	Lonchocarpol D	-9.068
222	Vogelin G	-9.061
223	Ficuisoflavone	-9.048
78	Fuscaflavanones A2	-9.043
201	Corylin	-8.971
40	Erythribyssin G	-8.966
137	Erylivingstone K	-8.965
193	Erysubin F	-8.951
138	2′,7-Dihydroxy-3′ -(3-methylbut-2- enyl)-2′′′,2′′′-dimethylpyrano [5′′,6′′:4′,5′]isoflavan	-8.946
73	Lonchocarpol A (Senegalensein)	-8.941
99	(2S)-5,7,5′-Trihydroxy-6′-prenyl-2′′-(4′′-hydroxyisopropyl)-3′′-hydroxy-dihydrofurano[1′′,3′′:4′,5′] flavanone	-8.941
90	2(S)-5,7-Dihydroxy- [2′′,2′′-(3′′,4′′-dihydroxy)- dimethylpyrano]-(5′′,6′′:3′,4′) flavanone	-8.933
174	Erythraddison IV	-8.922
24	Glabrol	-8.914
77	Fuscaflavanones A1	-8.887

91	2(S)-5,7-Dihydroxy-5′-prenyl [2′′,2′′-(3′′,4′′-dihydroxy)-dimethylpyrano]-(5′′,6′′:3′,4′) flavanone	-8.863
200	Erylatissin B	-8.861
51	6-Prenylabyssinone V	-8.845
241	Parvisoflavone B	-8.844
76	Lupinifolin	-8.841
72	Citflavanone	-8.838
181	5,4-Dihydroxy-2-methoxy-8-(3,3- dimethylallyl)-2,2-dimethylpyrano [5,6:6,7]isoflavanone	-8.833
243	Auriculasin	-8.821
244	Auriculatin	-8.814
123	Abyssinone B	-8.802
180	2,3-Dihydroauriculatin	-8.775
49	Abyssinone V	-8.774
66	Sigmoidin G	-8.762
34	Abyssinone-IV-4′-O-methyl ether	-8.756
236	Alpinumisoflavone	-8.722
260	Erymildbraedin B	-8.718
251	Derrone	-8.716
182	Glyasperin F	-8.711
254	Erysubin A	-8.704
257	5,7,4′-Trihydroxy-6-(3′′,3′′-dimethylallyloxiranylmethyl) isoflavone	-8.691
50	Abyssinone-V-4'-O-methyl ether	-8.689
1	6-Prenylapigenin	-8.688
136	Erylivingstone J	-8.685
41	Erythribyssin I	-8.684
160	Bidwillon B	-8.678
85	Abyssinoflavanone VI	-8.674
128	Eryzerin C	-8.674
140	Erypoegin B (Burttinol B)	-8.674
242	Warangalone (Scandenone)	-8.658
67	2(S)-5,5′,7-Trihydroxy-2′-prenyl- (2′′,2′′-dimethylpyrano)-(5′′,6′′:3′,4′)flavanone	-8.655
155	5-Deoxyglasperin F	-8.649
82	Abyssinin III	-8.643
129	4′,7-Dihydroxy-2′-methoxy-3′-(3- methylbut-2-enyl)isoflavan	-8.633
15	Vicenin-1	-8.628
124	Abyssinone C	-8.624
12	Isovitexin	-8.613
118	Abyssinone-VI-4-O-methyl ether	-8.611
63	Sigmoidin D	-8.608
59	Sigmoidin B	-8.594
84	Abyssinoflavanone V	-8.588
246	Robustone	-8.564
220	Vogelin E	-8.556
259	5,4-Dihydroxy-8-(3,3- dimethylallyl)-2-methoxyisopropylfurano[4,5:6,7] isoflavone	-8.555
202	Bidwillon C	-8.544
172	2,3-Dihydro-2′-hydroxyosajin	-8.535
60	3′-O-Methylsigmoidin	-8.533

36	5-Deoxyabyssinin II	-8.532
218	5-Hydroxy-3′′-hydroxy-2′′,2′′- dimethyldihydropyrano [5′′,6′′:3′,4′]isoflavone	-8.529
145	Eryvarin I	-8.525
57	Erycaffra F	-8.512
23	Isobavachin	-8.511
166	Eryvarin N	-8.508
168	Eryzerin B	-8.501
100	Addisoniaflavanone I	-8.499
80	Abyssinin I	-8.498
93	2(S)-5,5′,7-Trihydroxy-[2′′,2′′-(4′′- chromanone)- dimethylpyrano]-(5′′,6′′:3′,4′) flavanone	-8.495
255	Erysubin B	-8.494
95	(2S)-5,7-Dihydroxy-5′-methoxy-2′′- (4′′-hydroxyisopropyl)-dihydrofurano[1′′,3′′:3′,4′] flavanone	-8.487
179	5,3′-Dihydroxy-2′′,2′′-dimethylpyrano-[5,6:6,7]-2′′′,2′′′-dimethylpyrano[5,6:5,4] isoflavanone	-8.482
55	Burttinone	-8.463
247	M-Wi-2	-8.462
225	3′-(3-Methylbut-2-enyl) biochanin	-8.461
86	Abyssinoflavanone VII	-8.453
139	Erypoegin A (Burttinol C)	-8.435
4	Vogelin J	-8.433
205	6-Hydroxygenistein	-8.433
33	Abyssinone IV	-8.431
62	Sigmoidin C	-8.419
30	Abyssinone I	-8.413
135	2′-Methoxyphaseollinisoflavan	-8.411
117	Abyssinone VI	-8.408
28	5′-(2-Hydroxy-3-methylbut-3-enyl) abyssinone II	-8.401
159	Sigmoidin J	-8.393
219	4′-Hydroxy-5,7- dimethoxyisoflavone	-8.392
250	Osajin	-8.376
47	3′-Prenylnaringenin	-8.368
109	Erylivingstone F	-8.366
92	2(S)-5,5′,7-Dihydroxy-6′-prenyl [2′′,2′′-(3′′,4′′-dihydroxy)-dimethylpyrano]-(5′′,6′′:3′,4′) flavanone	-8.341
167	Eryvarin V	-8.337
249	4′,7-Dihydroxy-2′′,2′′- dimethylpyrano[5′′,6′′:5,6] isoflavone	-8.331
89	2(S)-5,7-Dihydroxy-5′-methoxy- [2′′,2′′-(3′′-hydroxy)-dimethylpyrano]-(5′′,6′′:3′,4′) flavanone	-8.329
39	2(S)-5′,7-Dihydroxy-[2′′,2′′-(3′′- hydroxy)-dimethylpyrano]-(5′′,6′′:3′,4′)flavanone	-8.327
18	Diosmetin-6-C-glucoside	-8.326
256	5,4′-Dihydroxy-2′-methoxy-8-(3,3- dimethylallyl)-2′′,2′′-dimethylpyrano[5,6:6,7]isoflavone	-8.315
173	Erythraddison III	-8.311
6	Atalantoflavone (Limonianin)	-8.309
148	Eriotrichin B (Bidwillon A)	-8.309
221	Vogelin F	-8.286

209	Isowighteone (3′-Isoprenylgenistein)	-8.283
203	Erythrinin A	-8.266
68	2(S)-5,5′,7-Trihydroxy- [2′′(5′′- hydroxy)-methylpyrano]-(5′′,6′′:3′,4′)flavanone	-8.258
37	7-Hydroxy-4′-methoxy-3′-(3- methylbut-2-enyl)flavanone	-8.256
54	Burttinonedehydrate	-8.255
69	2 (S)-5,7-Dihydroxy-3′-methoxy- [2′′(5′′-hydroxy)-methylpyrano]-(5′′,6′′:3′,4′)flavanone	-8.254
61	4′-O-Methylsigmoidin	-8.245
248	Erythgianin A	-8.243
58	Sigmoidin A	-8.242
152	Orientanol D	-8.238
42	Erylivingstone I	-8.235
233	5,4'-Dimethoxy-3'-prenylbiochanin A	-8.216
153	Orientanol F	-8.207
208	Lupiwighteone (8-Prenylgenistein)	-8.201
45	Eriodictyol	-8.197
224	5′-Prenylpratensein	-8.186
232	Schliebenone C	-8.181
253	Isochandalon	-8.175
48	Licoflavanone-4′-O-methyl ether	-8.153
65	Sigmoidin F	-8.153
102	Addisoniaflavanone III	-8.144
211	6,8-Diprenylorobol	-8.139
56	Erycaffra D	-8.138
191	8-Prenyldaidzein	-8.136
157	Sigmoidin H	-8.134
163	Eryzerin A	-8.132
122	Abyssinone A	-8.115
2	Luteolin	-8.113
217	5,4′-Dihydroxy-7-methoxy-3′-(3- methyl-2-hydroxybuten-3-yl) isoflavone	-8.106
154	5,2',4'-Trihydroxy-6-prenyl-2′′′,2′′′dimethyldihydropyrano[5′′′,6′′′] isoflavanone	-8.088
27	7,3′,4′-Trihydroxyflavanone	-8.086
207	Wighteone (Erythrinin B) (6-Prenylgenistein)	-8.086
164	Eryvarin B	-8.085
185	Erypoegin C (Vogelin B)	-8.075
141	Burttinol A	-8.072
25	Erythribyssin K	-8.069
46	Homohesperetin	-8.068
176	5,7,3′-Trihydroxy-4′-methoxy-6,5′di(γ,γ-dimethylallyl)isoflavanone	-8.066
11	Vitexin	-8.056
106	Erylivingstone C	-8.046
184	Vogelin A (Lysisteisoflavanone)	-8.043
175	2,3-Dihydropratensein	-8.042
35	Erylatissin C	-8.028
198	5-Deoxy-3'-prenylbiochanin A	-8.022
234	Laburnetin	-8.022
183	Licoisoflavanones	-8.015

116	Licoagrochalcone A	-8.006
237	4-O-Methylalpinumisoflavone	-8.006
245	Warangalone 4′-O-methyl ether	-8.004
107	Erylivingstone D	-8.003
87	2(S)-5′-(2-Hydroxy-3-methylbut-3- enyl)licoflavone-4′-O-methyl ether	-7.997
151	Erythribyssin J	-7.982
206	6,8-Diprenylgenistein	-7.962
171	Orientanol E	-7.959
105	Erylivingstone B	-7.955
104	Erylivingstone A	-7.953
194	Eryvarin S	-7.943
158	Sigmoidin I	-7.937
108	Erylivingstone E	-7.896
26	Liquiritigenin-5′-O-methyl ether	-7.893
43	Naringenin	-7.889
188	Vogelin D	-7.884
192	Neobavaisoflavone	-7.882
195	Erythraddison II	-7.881
186	Erypoegin D	-7.879
132	Eryvarin T	-7.877
161	2,3-Dihydro-2′-hydroxyneobavaisoflavanone	-7.855
196	2′,7-Dihydroxy-4′-methoxy-5′-(3- methylbut-2-enyl)isoflavone	-7.833
199	Erylatissin A	-7.833
149	Prostratol C	-7.816
214	5,2′,4′-Trihydroxy-7-methoxy-5′-(3- methylbuten-2-yl)isoflavone	-7.798
133	Erythribidin A	-7.795
44	Isosakuranetin	-7.791
143	Bidwillol A	-7.791
22	Liquiritigenin	-7.776
111	Erylivingstone H	-7.767
142	7,4′-Dihydroxy-2′,5′- dimethoxyisoflav-3-ene	-7.737
258	Erymildbraedin A	-7.737
110	Erylivingstone G	-7.722
239	Indicanine C	-7.679
156	5-Deoxylicoisoflavanones	-7.676
150	Erythribyssin E	-7.659
230	Schliebenone A	-7.656
147	Vestitone	-7.652
228	5′-Formylpratensein	-7.638
144	Eryvarin H	-7.615
146	Eryvarin O	-7.598
229	5,7-Dihydroxy-4′-methoxy-3′-(2,3- dihydroxy-3-methylbutyl) isoflavone	-7.593
216	3′-Formyl-5,4′-dihydroxy-7- methoxyisoflavone	-7.588
114	Isobavachalcone	-7.578
226	Piscerythrinetin	-7.561
5	Carpachromene	-7.546
190	Daidzein	-7.519
19	3,7,4′-Trihydroxyflavone	-7.516
227	2′-Hydroxy-5′-methoxybiochanin A	-7.506

120	3-O-Methylbutein	-7.495
215	Cajanin	-7.483
213	5,4′-Dihydroxy-7-methoxy-3′-(3- methylbuten-2-yl)isoflavone	-7.481
126	2,4,4'-Trihydroxychalcone	-7.471
177	(R)-saclenone	-7.468
204	Genistein	-7.413
115	Isoliquiritigenin	-7.335
238	5,4′-Dimethoxy alpinumisoflavone	-7.319
169	Erycaffra E	-7.315
240	Indicanine E	-7.221
3	Vogelin C	-7.094
8	Salvigenin	-7.093
119	Butein	-7.051
10	Sinensetin	-7.023
9	Tetramethylisoscutellarein	-6.966
112	Hamiltone A	-6.907
170	5,7-Dihydroxy-2′,4′,5′trimethoxyisoflavanone	-6.902
113	6-Methoxyhamiltone A	-6.851
127	6′-Hydroxy-2′,3′,4′,4- tetramethoxychalcone	-6.838
21	Kaempferol-3-O-β-ᴅglucopyranosyl- (1 →2)-β-ᴅ-glucopyranoside	-5.542
13	Isovitexin-2′′-β-ᴅ- glucopyranoside	-1.257
287	4′-Hydroxyisoflavone-7-O-α-L-rhamnosyl/ (1 →6)-β-ᴅ-glucopyranoside	-10.36
339	Erypoegin J	-10.15
354	Sigmoidin K	-9.941
341	Erysubin E	-9.825
333	Demethylerystagallin A	-9.79
345	Erycristagallin	-9.771
325	Erythribyssin L	-9.469
351	Erythribyssin O	-9.377
355	Isosojagol	-9.275
97	(2S)-5,7-Dihydroxy-5′-prenyl-2′′- (4′′-hydroxyisopropyl)- 3′′-hydroxy-dihydrofurano [1′′,3′′:3′,4′]flavanone	-9.2
334	Erystagallin B	-9.126
32	Abyssinone III	-9.12
264	Erysenegalensein F	-9.061
347	Erypoegin H	-9.058
83	Abyssinoflavanone IV	-9.05
252	Isoderrone	-9.04
286	4′-Hydroxyisoflavone-7-O-β-ᴅ-glucopyranoside	-9.021
332	Erystagallin A	-8.937
134	Phaseollinisoflavan	-8.89
278	Vogelin I	-8.888
267	Erysenegalensein L	-8.865
371	Erythribyssin F	-8.824
360	Erysenegalensein J	-8.812
350	Eryvarin W	-8.756
344	Hydroxycristacarpone	-8.754
53	5-Hydroxysophoranone	-8.74

38	2(S)-5′,7-Dihydroxy-[2′′,2′′-(3′′- hydroxy)-dimethylpyrano]-(5′′,6′′:3′,4′)flavanone	-8.71
349	Eryvarin E	-8.708
319	Orientanol C	-8.706
275	Hydroxyerythrinin C	-8.701
268	Erysenegalensein M	-8.7
366	Eryvarin Q	-8.689
189	Auriculatin 4'-O-glucoside	-8.66
17	Isoorientin	-8.65
308	Erybraedin B	-8.649
279	Eriotriochin	-8.63
294	1-Methoxyerythrabyssin II	-8.607
314	Erylysin A	-8.596
324	Folitenol	-8.585
323	Phaseollin	-8.547
346	Erypoegin E	-8.526
276	8-Prenylerythrinin C (Isosenegalensein) (Euchrenone b10)	-8.518
274	Erythrinin C	-8.517
375	Kanzonol U (Glabrocoumarone A)	-8.504
315	Erylysin B	-8.501
311	Erybraedin E	-8.484
270	Erysenegalensein O	-8.477
266	Erysenegalensein K	-8.468
277	Vogelin H	-8.43
272	Erypoegin K	-8.425
265	Erysenegalensein G	-8.417
300	Erythribyssin C	-8.411
81	Abyssinoflavanone II (Abyssinin II)	-8.4
284	2,3-Dehydrokievitone	-8.383
31	Abyssinone II	-8.36
71	Erysenegalone (Erythrisenegalone)	-8.36
293	Erythrabyssin II	-8.36
280	Erythraddison I	-8.357
310	Erybraedin D	-8.35
298	Eryvarin K	-8.347
290	Sophorapterocarpan A (Homoedudiol)	-8.328
335	Eryzerin E	-8.314
321	Isoneorautenol	-8.283
307	Erybraedin A	-8.281
292	1-Methoxy phaseollidin	-8.279
322	8-Methoxyneorautenol	-8.275
125	Abyssinone D	-8.27
330	Fuscacarpan A	-8.269
318	Orientanol B	-8.265
273	Senegalensin	-8.241
373	Addisofuran A	-8.238
331	Cristacarpin (Erythrabissin I)	-8.203
374	Addisofuran B	-8.209
317	Shinpterocarpin	-8.191

29	7-Hydroxy-4′-methoxy-3′-(3- hydroxy-3-methyl-trans-but-1- enyl)-5′-(3-methylbut-2-enyl) flavanone	-8.18
295	Calopocarpin	-8.168
121	5-Prenylbutein	-8.15
342	Eryvarin A	-8.149
362	2-(5′-Hydroxy-3′′-methoxyphenyl)- 6-hydroxy-5-methoxybenzofuran	-8.136
101	Addisoniaflavanone II	-8.13
263	Isoerysenegalensein E (Lysisteisoflavone)	-8.124
297	Eryvarin J	-8.12
309	Erybraedin C	-8.115
369	Burttinol D	-8.099
187	Erypoegin G	-8.08
353	4-Hydroxycoumasterol	-8.059
352	Coumasterol	-8.044
356	Erythribyssin N	-8.043
299	Erythribyssin B	-8.04
271	5,7,4′-Trihydroxy-6-(2′′-hydroxy3′′-methylbut-3′′enyl) isoflavone	-8.038
296	3,9-Dihydroxy-4-prenylpterocarpan	-8.013
285	8-Prenylluteone	-8.012
372	2′-O-Demethylbidwillol B	-8.008
340	Orientanol A	-8.002
363	Vignafuran	-7.968
291	Phaseollidin	-7.965
303	Erycristin	-7.937
162	Eryvellutinone	-7.91
368	Glyinflanin H	-7.887
316	Erylysin C	-7.883
312	Erybraedin F	-7.876
261	Erysenegalensein D	-7.873
103	5,7-Dihydroxy-3′,4′-dimethoxy-5′- (3-methylbut-2-enyl)flavanone	-7.86
212	3′-O-Methylorobol	-7.86
283	7-O-Methylluteone	-7.86
320	Neorautenol	-7.858
364	Eryvarin L	-7.843
367	Eryvarin U	-7.831
348	Eryvarin D	-7.825
231	Schliebenone B	-7.81
328	Erysubin C	-7.792
361	Latissimbenzofuran	-7.772
269	Erysenegalensein N	-7.766
289	Demethylmedicarpin	-7.754
197	Calycosin	-7.74
343	Erythribyssin A	-7.718
358	Indicanine B	-7.713
338	Erypoegin I	-7.693
370	Erypoegin F	-7.687
357	Indicanine A	-7.663
313	Erystagallin C	-7.658
262	Erysenegalensein E	-7.656
165	Eryvarin M	-7.65

337	Fuscacarpan C	-7.616
336	Fuscacarpan B	-7.613
306	Dolichins A and B	-7.588
365	Eryvarin P	-7.554
301	Medicarpin	-7.512
304	3-Hydroxy-10-(3-hydroxy-3- methylbutyl)-9- methoxypterocarpan	-7.506
305	3-Hydroxy-10-(2,3-dihydroxy-3- methylbutyl)-9- methoxypterocarpan	-7.35
359	Robustic acid	-7.312
377	Eryvarin R	-7.275
281	Panchovillin	-7.231
376	Erythribyssin H	-7.225
282	7-Demethylrobustigenin	-7.194
302	Sandwicensin	-7.069
326	Erythribyssin D	-6.785
329	Erysubin D	-6.422
327	Erythribyssin M	-5.337
235	Indicanine D	-5.26
288	Derriscanoside B	-5.075
378	Bis-Sigmodiol	-1.257
